# Macrophage migration inhibitory factor from nematode parasite as a novel approach to combat obesity and its metabolic complications

**DOI:** 10.3389/fimmu.2026.1742993

**Published:** 2026-02-05

**Authors:** Mi-Kyung Park, MinKyoung Cho, Hak Sun Yu

**Affiliations:** 1 Department of Parasitology and Tropical Medicine, School of Medicine, Pusan National University, Yangsan, Republic of Korea; 2 Department of Parasitology and Tropical Medicine, and Institute of Medical Science, Gyeongsang National University College of Medicine, Jinju, Republic of Korea; 3 Department of Convergence Medical Science, Gyeongsang National University, Jinju, Republic of Korea; 4 Research Institute for Convergence of Biomedical Science and Technology, Pusan National University Yangsan Hospital, Yangsan, Republic of Korea

**Keywords:** *Anisakis simplex*, immunomodulation, inflammation, macrophage migration inhibitory factor, obesity, rAs-MIF

## Abstract

**Objective:**

Obesity is a complex chronic disease characterized by excessive fat accumulation, dysregulation of energy homeostasis, and associated mild inflammation, significantly increasing the risk of metabolic diseases, cardiovascular diseases, and other chronic diseases. Addressing this requires innovative approaches targeting the underlying mechanisms. This study investigates the therapeutic effects of *Anisakis simplex-*derived macrophage migration inhibitory factor (1) on adipogenesis, lipid metabolism, and inflammation in high-fat diet (2)-induced obesity.

**Methods:**

We evaluated the effects of rAs-MIF on adipocyte differentiation, lipid droplet formation, and expression of adipogenic and inflammatory genes in 3T3-L1 cells. In addition, we investigated the effects of orally administered rAs-MIF on lipid accumulation, glucose metabolism, serum biochemical markers, and immune regulation in mice fed a 45% high-fat diet.

**Results:**

rAs-MIF dose-dependently reduced lipid droplet size and triglyceride accumulation, inhibited adipogenesis and inflammatory gene expression, and upregulated adiponectin levels in 3T3-L1 cells. In HFD-fed mice, rAs-MIF decreased body weight gain, fat mass, and serum lipid levels, enhanced glucose tolerance, and activated interscapular brown adipose tissue while suppressing epididymal white adipose tissue lipid synthesis. Moreover, it modulated the immune balance by promoting the polarization of anti-inflammatory macrophages and reducing the production of pro-inflammatory cytokines.

**Conclusion:**

rAs-MIF demonstrates a dual regulatory effect on adipogenesis and metabolism, highlighting its potential as a therapeutic candidate for obesity.

## Highlights

- rAs-MIF inhibits adipogenesis by reducing lipid accumulation and adipogenic gene expression.
- rAs-MIF reduces body weight gain, fat mass, and serum lipids in HFD-fed mice.
- rAs-MIF activates iBAT and suppresses eWAT lipid accumulation.
- rAs-MIF shifts macrophage polarization toward anti-inflammatory M2 phenotypes.
- rAs-MIF demonstrates therapeutic potential for obesity and related inflammation.

## 1 Introduction

Obesity is a risk factor for chronic diseases, characterized by an increase in both the size and number of adipocytes, which differentiate from precursor cells (2–5). This condition is closely associated with metabolic disorders, including type 2 diabetes, cardiovascular diseases, and non-alcoholic fatty liver disease (6–8). Despite extensive research into obesity therapies, effective interventions with minimal side effects remain a challenge (9).

Adipose tissue plays a vital role in energy storage and metabolic regulation. Dysregulation of white adipose tissue (10), which primarily stores energy, and brown adipose tissue (7), which dissipates energy as heat, contributes significantly to the development and progression of obesity and related metabolic syndromes (11, 12). Targeting the mechanisms underlying adipocyte differentiation and metabolism has emerged as a promising strategy for obesity management. The enlargement of WAT through an increase in white adipocyte number, macrophage infiltration, and fibrosis disrupts hormonal homeostasis (12, 13). This process leads to the release of inflammatory cytokines and adipokines, which interfere with normal energy balance and contribute to the development of metabolic syndrome (14). Adipogenesis, the differentiation process by which pre-adipocytes become fully mature adipocytes, is crucial for the development and functional regulation of adipose tissue. This process is marked by the progressive accumulation of intracellular lipids in maturing adipocytes. Key adipogenic transcription factors such as CCAAT-enhancer-binding protein alpha (C/EBPα), fatty acid binding protein 4 (aP2) and peroxisome proliferator-activated receptor gamma (PPARγ), control promoting adipocyte differentiation and enhancing the expression of adipokines that further facilitate adipogenesis (11, 15). Given the important role of adipocytes in regulating adipokine secretion and energy metabolism, understanding the molecular mechanisms underlying adipogenesis is essential for developing effective anti-obesity therapies. M2 macrophages play a crucial role in maintaining adipose tissue homeostasis by secreting anti-inflammatory cytokines such as IL-10 and TGF-β (16). In addition to their immunomodulatory functions, M2-polarized macrophages support healthy adipose tissue remodeling and enhance insulin sensitivity by regulating the metabolic environment and promoting the thermogenic activity of adipocytes (17, 18). Therefore, polarizing macrophages to the M2 phenotype is emerging as a promising therapeutic strategy for managing obesity-related metabolic disorders (19).

Parasitic helminths are known as the strongest natural inducers of the type 2 immune response, which can modulate inflammatory pathways (20, 21). Furthermore, chronic parasitic infections are often accompanied by the activation of regulatory T cells (T_regs_), which secrete IL-10 and TGF-β to suppress excessive inflammation and maintain immune tolerance (22, 23). This mechanism is particularly important, as increased IL-10 and TGF-β levels were observed in obese mice treated with rAs-MIF, suggesting the possible involvement of T_regs_ in mediating the immunomodulatory effects (24, 25). Several epidemiological and experimental studies have demonstrated an inverse correlation between helminth infection and metabolic syndromes, including obesity. This phenomenon has spurred interest in exploring helminth-derived molecules for therapeutic applications in metabolic diseases (2, 5, 26, 27). One such molecule, *Anisakis simplex*-derived macrophage migration inhibitory factor (1), has shown potent anti-inflammatory properties. Recombinant rAs-MIF (1) suppresses excessive inflammation by inhibiting macrophage activity and reducing pro-inflammatory mediators such as TNF-α and IL-1β while promoting the release of anti-inflammatory cytokines like IL-10 (1, 24, 25). These immunomodulatory effects have been explored in autoimmune and metabolic disease, highlighting its promising therapeutic potential (28). Despite the immunological functions of rAs-MIF, its specific impact on obesity, adipogenesis, or fat metabolism has not been extensively studied. With the growing interest in the central role of rAs-MIF in macrophage polarization and inflammation, future research may indirectly explore its implications for obesity management, particularly since inflammation is a key aspect of obesity-related metabolic disorders.

Therefore, in this study, we aimed to explore the potential of rAs-MIF as a novel therapeutic agent for obesity. To this end, we assessed its impact on adipocyte function and the expression of lipid metabolism-related genes in epididymal WAT (eWAT) and interscapular BAT (iBAT) in both cell-based adipogenesis model and HFD-induced obese mouse model.

## 2 Materials and methods

### 2.1 Preparation of rAs-MIF

In our previous study, rAs-MIF was cloned into the pET-28a expression vector (Novagen, Darmstadt, Germany) and purified following an established protocol (1, 24).

### 2.2 Cell culture and adipocyte differentiation

The murine 3T3-L1 pre-adipocyte cell line was purchased from ATCC (Manassas, VA, USA). The cells were incubated in Dulbecco’s modified Eagle’s medium (DMEM; Gibco, USA) containing 10% bovine calf serum (ATCC) and 1% penicillin/streptomycin (P/S). Differentiation was initiated on day 2 after confluence, and then stimulated with DMEM containing glucose (4.5 g/L) and supplemented with 10% fetal bovine serum and 1% P/S, 1 μM dexamethasone, 0.5 mM isobutylmethylxanthine, 1 g/mL insulin, and either 1, 5, or 10 μg of rAs-MIF or phosphate-buffered saline (PBS) as a negative control for 48 hours. After differentiation, the medium was replaced with DMEM containing 1 μg/mL insulin for an additional 2 days. The medium was refreshed every 48 hours from day 2 to day 12. The differentiation-induced adipocytes in the absence of rAs-MIF served as the positive control, representing fully differentiated adipocytes used for comparison with the experimental groups.

### 2.3 Oil red O staining

Differentiated 3T3-L1 cells were stained with Oil red O as described previously (29). Briefly, the 3T3-L1 cells were washed with PBS and fixed in 4% formalin (Sigma-Aldrich, St. Louis, MO, USA). Then the cells were washed with distilled water and subsequently incubated with 60% isopropanol (Sigma-Aldrich). Following this step, the cells were stained with Oil Red O solution (0.5 g/L; Sigma-Aldrich) for 30 minutes at room temperature and thoroughly washed with distilled water. Microscopic images were captured using an optical microscope. The retained Oil Red O dye was extracted with pure isopropanol to quantify lipid accumulation, and absorbance was measured at 490 nm.

### 2.4 Ethic approval

All animal experiments were performed according to the National Institutes of Health Guide for the Care and Use of Laboratory Animals and protocols approved by the Pusan National University-Institutional Animal Care and Use Committee (PNU-IACUC; approval No.PNU-2022-0182; Obesity improvement effect by parasitic nematode type II MIF).

### 2.5 Obesity mouse model and As-MIF administration

Six-week-old male C57BL/6 mice were purchased from Samtako (Gyeonggi-do, Republic of Korea). Mice were housed in a controlled environment (temperature: 25 ± 2°C, humidity: 50-60%, 12-hour light/dark cycle). All experiments were performed using male mice to minimize variability associated with hormonal cycles. It is acknowledged that sex hormones can influence metabolic and immune responses, including MIF expression and macrophage polarization; therefore, future studies using female mice will be necessary to determine whether rAs-MIF exerts comparable immunometabolic effects across sexes (30, 31).

After a one-week acclimatization period, the mice were randomly assigned to three groups, a normal diet group, a high-fat diet (HFD) group, and a HFD with rAs-MIF administered group (n = 6 per group). Mice were fed a normal diet or a 45% HFD (New Brunswick, NJ, USA) to induce obesity for 8 weeks. From 4 to 8 weeks, rAs-MIF (10 and 50μg) was administered orally every other day. The normal diet and 45% HFD were continuously provided to the respective groups for the duration of the experiment.

### 2.6 Whole-body composition, body weight dynamics, and food intake

Whole-body composition, including body fat and lean mass, was quantitatively measured using magnetic resonance analysis (Minispec Analyzer LF50, Bruker, Germany) in conscious animals one day prior to the termination of the experiment. Body weight was recorded weekly to evaluate trends in weight gain across groups. Food intake was determined by weighing the remaining food in the cages at the end of each week. Food efficiency was calculated as the ratio of body weight gain to total food intake over the same period, expressed as the food efficiency ratio (FER = total body weight gain (g)/total food intake (g)).

### 2.7 Oral glucose tolerance tests

For OGTT, fasted mice for 16 hours were orally injected with 20% glucose solution at a dose of 2 g/kg fasting body weight. Blood glucose levels were measured from tail vein samples at 0, 15, 30, 60, and 120 minutes post-administration using a glucometer (Accu-Chek, Roche Diagnostics, Mannheim, Germany).

### 2.8 Assessment of adipose tissue distribution

eWAT and iBAT were excised and weighed to assess fat distribution. The weight of each tissue was normalized to the total body weight. Gene expression levels related to lipid metabolism and inflammation in eWAT and iBAT were analyzed using quantitative PCR.

### 2.9 Serum biochemistry

Plasma total cholesterol (TC), glucose concentration (GLU), and lactate dehydrogenase (LDH) levels were quantified using a Fuji Dri-Chem system (FUJIFILM Corp., Tokyo, Japan), which is based on dry chemistry technology.

### 2.10 Histological analysis

Liver tissues were isolated from mice, fixed in 4% formaldehyde, and embedded in paraffin. The paraffin-embedded liver sections were then stained with hematoxylin and eosin (H&E). Following staining, the sections were examined under a microscope for histopathological analysis.

### 2.11 RNA extraction and cDNA synthesis

Total RNA was isolated using TRIzol reagent (Qiagen, Hilden, Germany) according to the manufacturer’s protocol. The concentration and purity of RNA were assessed using a NanoDrop 2000 spectrophotometer (Thermo Fisher Scientific, Waltham, MA, USA). Complementary DNA (cDNA) was synthesized from 2 μg of total RNA using M-MLV Reverse Transcriptase (Promega, Madison, WI, USA) and oligo(dT) primers following the manufacturer’s protocol. Quantitative reverse transcription-time PCR was performed using SYBR™ Select Master Mix (Thermo Fisher Scientific, Waltham, MA, USA) in a LightCycler 96 Real-Time PCR System (Thermo Fisher Scientific). Relative gene expression was calculated using the 2^-ΔΔCt method, with GAPDH used as a reference gene. The primer sequences are listed in **Table 1**.

**Table 1:** Primer sequences for real-time PCR.

Primer	Sequence
GAPDH-for^*^	5’- CAT CAC TGC CAC CCA GAA GAC TG -3’
GAPDH-rev	5’- ATG CCA GTG AGC TTC CCG TTC AG -3’
C/EBPα-for	5’- CAA AGC CAA GAA GTC GGT GGA CAA -3’
C/EBPα-rev	5’- TCA TTG TGA CTG GTC AAC TCC AGC-3’
PPARγ-for	5’- GCC TCC TGG TGA CTT TAT GGA-3’
PPARγ-rev	5’- GCA GCA GGT TGT CTT GGA TG-3’
leptin-for	5’- TGG CTT TGG TCC TAT CTG TC -3’
leptin-rev	5’- TCC TGG TGA CAA TGG TCT TG -3’
ap2-for	5’- TGA AAT CAC CGC AGA CGA CAG G -3’
ap2-rev	5’- GCT TGT CAC CAT CTC GTT TTC TC -3’
adiponectin-for	5’- TGA CGA CAC CAA AAG GGC TC -3’
adiponectin-rev	5’- ACC TGC ACA AGT TCC CTT GG -3’
Asc-1-for	5’- TTC CAG GAA GGC TTT GAT GGC G -3’
Asc-1-rev	5’- CCA AAG TCT TCC TCT GTG AGG AG -3’
Hoxc9-for	5’- CAG CAA GCA CAA AGA GGA GAA GG -3’
Hoxc9-rev	5’- AGT TCC AGC GTC TGG TAC TTG G -3’
GLUT-4-for	5’- GGT GTG GTC AAT ACG GTC TTC AC -3’
GLUT-4-rev	5’- AGC AGA GCC ACG GTC ATC AAG A -3’
MGL2-for	5’- CGA GAC TTG AGC CAG AAG GTG A -3’
MGL2-rev	5’- GCC TTC AAG TCT GTC TCC AGC T -3’
CD206-for	5’- GTT CAC CTG GAG TGA TGG TTC TC -3’
CD206-rev	5’- AGG ACA TGC CAG GGT CAC CTT T -3’
PPARα-for	5’- ACC ACT ACG GAG TTC ACG CAT G -3’
PPARα-rev	5’- GAA TCT TGC AGC TCC GAT CAC AC -3’
UCP-1-for	5’- GCT TTG CCT CAC TCA GGA TTG G -3’
UCP-1-rev	5’- CCA ATG AAC ACT GCC ACA CCT C -3’

For, forward; rev, reverse.

### 2.12 Isolation of stromal vascular fraction from adipose tissue

Stromal vascular fractions (SVFs) were isolated from eWAT for the analysis of macrophage populations. eWAT was excised and minced into small fragments in ice-cold PBS. The tissue was digested with 0.2% collagenase type I (Sigma-Aldrich, St. Louis, MO, USA) in PBS at 37°C for 30 minutes with gentle shaking. The digested tissue was filtered through a 100 μm cell strainer to remove undigested fragments, and the resulting suspension was centrifuged at 300 × g for 5 minutes to separate the SVF pellet, the supernatant was removed and the pellet was resuspended in ACK lysis buffer.

### 2.13 Analysis of cell profile and cytokine production

Macrophage polarization and cytokine production were analyzed to profile immune responses. Stromal vascular fraction (SVF) cells were stained with antibodies against F4/80 (BL1/FITC, clone BM8), CD11b (VL1/BV421, clone M1/70), CD45 (BL1/PE-Cy7, clone ER-MP23), CD11c (BL2/APC-Cy7, clone N418), and CD206 (BL1/PE, clone MEL-14) (all from eBioscience, San Diego, CA, USA). Data were acquired on a BD FACSCanto II cytometer (BD Biosciences) using the indicated detector channels (BL1, BL2, and VL1). Compensation was performed post-acquisition in FlowJo software (BD biosciences) using an unstained control and single-positive cell populations identified within the fully stained samples to generate the spillover matrix. Macrophage populations were identified as CD11b^+^F4/80^+^ cells, gated within the CD45^+^ population, after sequential exclusion of debris and doublets. M1 was defined as CD11c^+^ and M2 as CD206^+^ within the F4/80^+^ population.

The cytokine levels of TNF-α, IFN-γ, IL-1β, IL-6, IL-10, and TGF-β in serum were quantified using ELISA kits (eBioscience, San Diego, CA, USA), according to the manufacturer’s protocol.

### 2.14 Statistical analysis

All data were analyzed using Prism 6 (GraphPad Prism, La Jolla, CA, USA). Mean ± standard deviation (19) was calculated, and significant differences were determined using the Student’s t-test or one-way analysis of variance (ANOVA) with Dunnett’s multiple comparisons post-test comparing all groups with the control group. A p-value of < 0.05 was considered statistically significant.

## 3 Results

### 3.1 rAs-MIF suppresses adipogenic differentiation of preadipocyte 3T3-L1 cells

**Figure 1:**
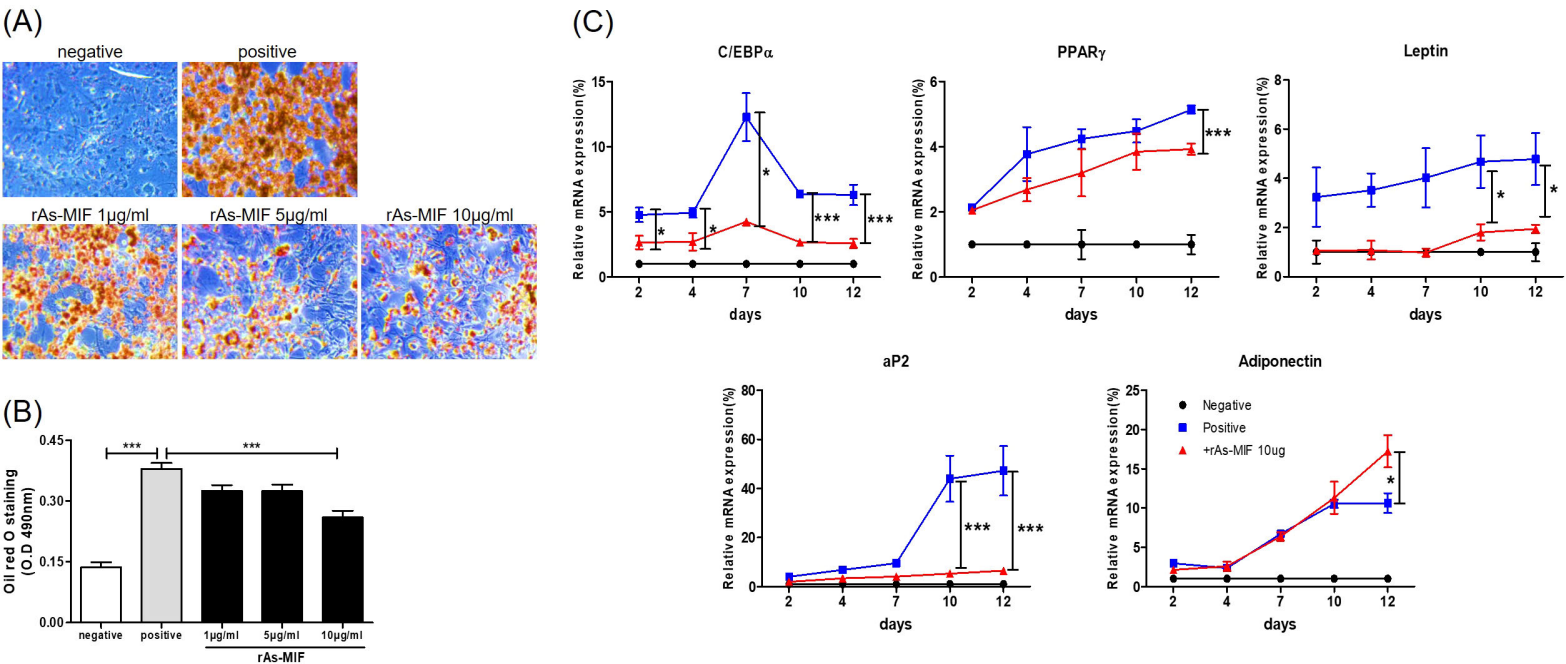
Influence of parasite-derived rAs-MIF on differentiation in 3T3-L1 cells. **(A)** Oil Red O staining images of 3T3-L1 adipocytes. The positive control is fully differentiated adipocytes in the absence of rAs-MIF, and the negative control is undifferentiated preadipocytes. **(B)** Quantitative analysis of lipid droplet accumulation and triglyceride content. **(C)** Expression levels of C/EBPα, PPARγ, leptin, Ap2, and adiponectin were evaluated by real-time PCR during adipocyte differentiation (n = 5/group, 3 independent experiments, *p < 0.05, ***p < 0.001).

To investigate the effect of rAs-MIF on adipocyte hypertrophy, we assessed the size of lipid droplet formation in 3T3-L1 mouse adipocytes differentiated for 12 days in a medium containing increasing glucose concentrations. Representative images of lipid droplets stained with Oil Red O (**Figures 1A, B**) showed that the positive control group exhibited significantly larger lipid droplets compared to the undifferentiated negative control. In contrast, lipid accumulation and triglyceride content were significantly reduced in a dose-dependent manner in the rAs-MIF-treated group (**Figures 1A, B**). To assess the effects of rAs-MIF on adipocyte differentiation, we analyzed the expression of key regulatory genes in 3T3-L1 cells. Compared with the positive control (differentiated adipocytes not treated with rAs-MIF or PBS), rAs-MIF treatment significantly decreased the mRNA levels of key adipogenic transcription factors C/EBPα and PPARγ, as well as their downstream target genes leptin and aP2, throughout the differentiation process. In contrast, the expression of adiponectin, an insulin-sensitive adipokine that promotes metabolic activity, was significantly increased on day 12, the final stage of differentiation. These results suggest that rAs-MIF inhibits adipocyte differentiation while simultaneously promoting the expression of metabolic genes. Mechanistically, this dual regulation suggests that rAs-MIF inhibits lipid droplet maturation by interfering with the C/EBPα–PPARγ transcriptional cascade, while upregulating the adiponectin-mediated pathway, which contributes to enhanced metabolic and anti-inflammatory activity within adipocytes. Collectively, these results highlight the potential of rAs-MIF as a regulator of adipogenesis and adipocyte metabolic function (**Figure 1C**).

### 3.2 rAs-MIF improves the key obesity parameters in HFD-induced obese animal model

**Figure 2:**
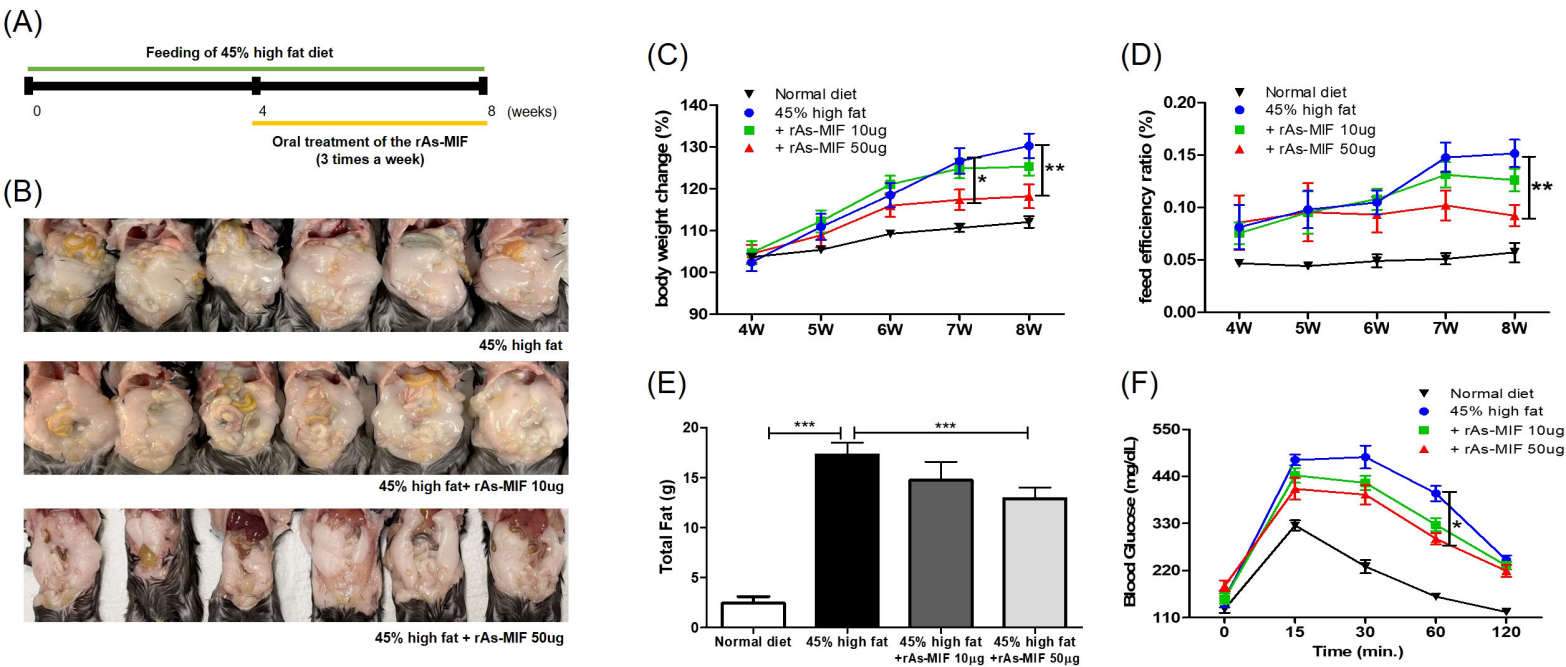
Effect of rAs-MIF on obesity phenotypes in 45% HFD-fed mice. **(A)** Schematic of rAs-MIF treatment in a 45% HFD-induced obesity model. **(B)** Representative images of mice and eWAT from each group after the terminal experiment. **(C)** Body weight, **(D)** Food efficiency ratio (45) = increased body weight (g)/food intake (g), and **(E)** Total fat weight using LF50. **(F)** The blood glucose level in oral glucose tolerance tests in mice. **(G)** Epididymal fat mass to total fat mass ratio by group. **(H)** Interscapular brown adipose tissue mass to total fat mass ratio by group. (n = 6/group, 3 independent experiments, *p < 0.05, **p < 0. 01, ***p < 0.001).

To evaluate the therapeutic potential of recombinant arsenic-myelin (rAs-MIF) *in vivo*, we administered the recombinant protein orally to high-fat diet-induced obese mice, and monitored various metabolic parameters (**Figures 2A, B**). As expected, the high-fat diet-fed group significantly increased body weight gain (**Figure 2C**) and feed efficiency (**Figure 2D**). However, rAs-MIF administration significantly attenuated these obesity-related changes. Notably, total fat mass was measured using magnetic resonance imaging (MRI) one day before the end of the study. rAs-MIF-treated mice showed a significant decrease in fat mass compared to the high-fat diet-fed control group (**Figure 2E**). In addition, the glucose concentration in blood calculated by the OGTT was significantly lower in rAs-MIF-treated groups (10 μg and 50 μg) than in the HFD group, indicating improved glucose tolerance by rAs-MIF treatment (**Figure 2F**).

### 3.3 rAs-MIF regulates iBAT activation and lipid metabolism in HFD-fed mice

**Figure 3:**
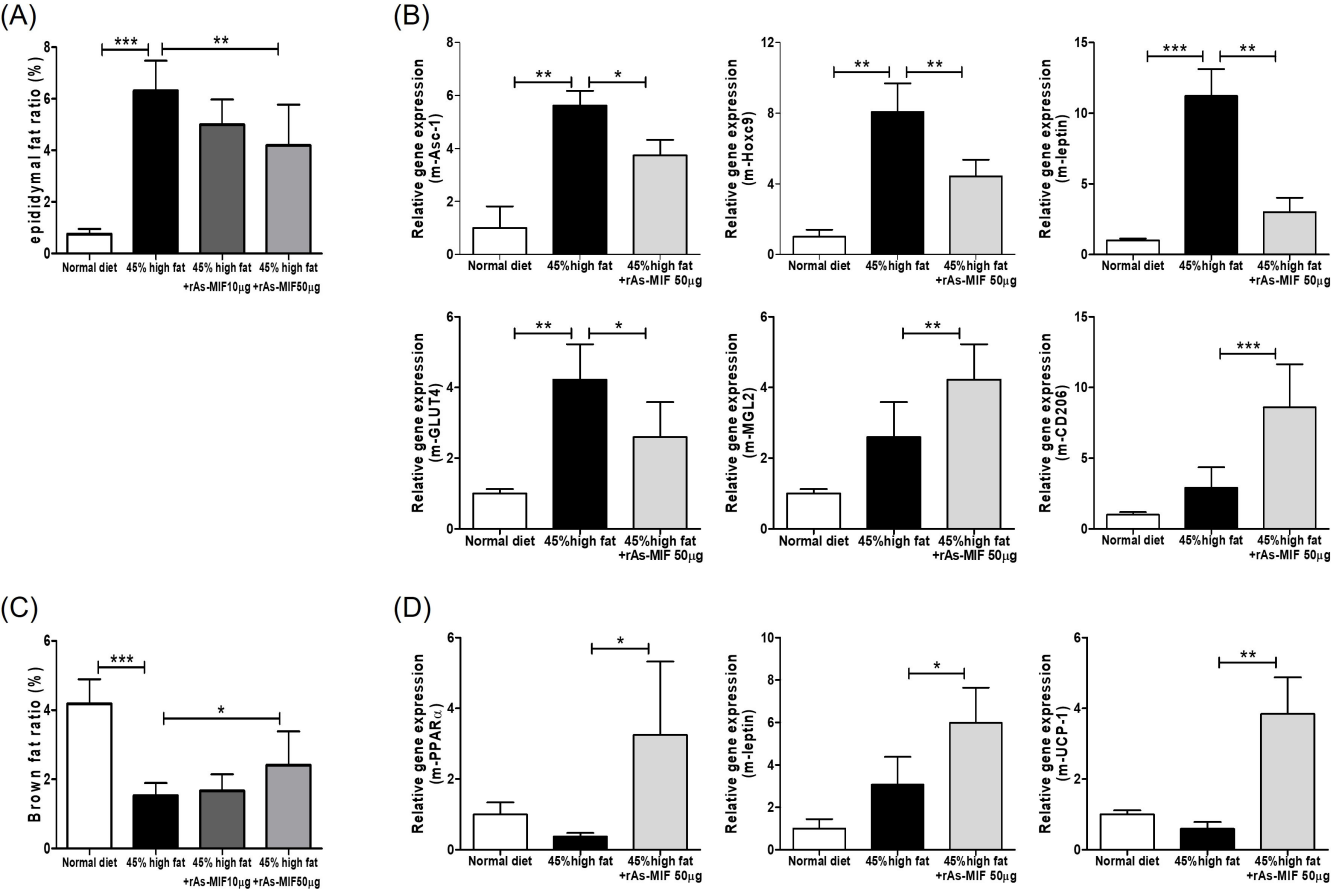
Effects of rAs-MIF on lipid metabolism and adipose tissue gene expression in mice with 45% HFD-induced obesity. **(A)** The ratio of epididymal white adipose tissue (eWAT) to total body weight. **(B)** Relative mRNA expression levels of adipogenic and lipid metabolism-related genes (Asc-1, Hoxc9, leptin, GLUT4, MGL2, and CD206) in eWAT. **(C)** The ratio of brown adipose tissue (BAT) to total body weight. **(D)** Relative mRNA expression levels of metabolic and thermogenic genes (PPARα, leptin, and UCP-1) in interscapular brown adipose tissue (iBAT). (n = 6/group, 3 independent experiments, *p < 0.05, **p < 0. 01, ***p < 0.001).

Following the observation that rAs-MIF attenuates obesity-induced phenotypes, we further investigated its site-specific effects on adipose tissue remodeling and lipid metabolism. Analysis of fat mass distribution revealed that rAs-MIF treatment significantly reduced lipid accumulation in epididymal white adipose tissue (eWAT) (**Figure 3A**) while concurrently enhancing the metabolic activation of interscapular brown adipose tissue (iBAT) (**Figure 3C**). To elucidate the molecular mechanisms underlying these tissue-specific responses, we analyzed the expression of key metabolic and adipogenic regulators. In eWAT, rAs-MIF markedly suppressed the mRNA levels of pro-adipogenic and inflammatory markers, including PPARγ, C/EBPα, and leptin, suggesting an inhibition of adipocyte hypertrophy and lipid synthesis (**Figure 3B**). In contrast, rAs-MIF administration upregulated the expression of PPARγ, UCP-1, and PPARα in iBAT, indicating an induction of lipid oxidation and thermogenic activity (**Figure 3D**). These results suggest that rAs-MIF contributes to overall metabolic improvement by differentially regulating adipogenesis in white adipose tissue and metabolic activation and energy expenditure in brown adipose tissue depending on the adipose tissue site.

### 3.4 rAs-MIF attenuates hyperlipidemia and hepatic steatosis in an HFD-induced obesity model

**Figure 4:**
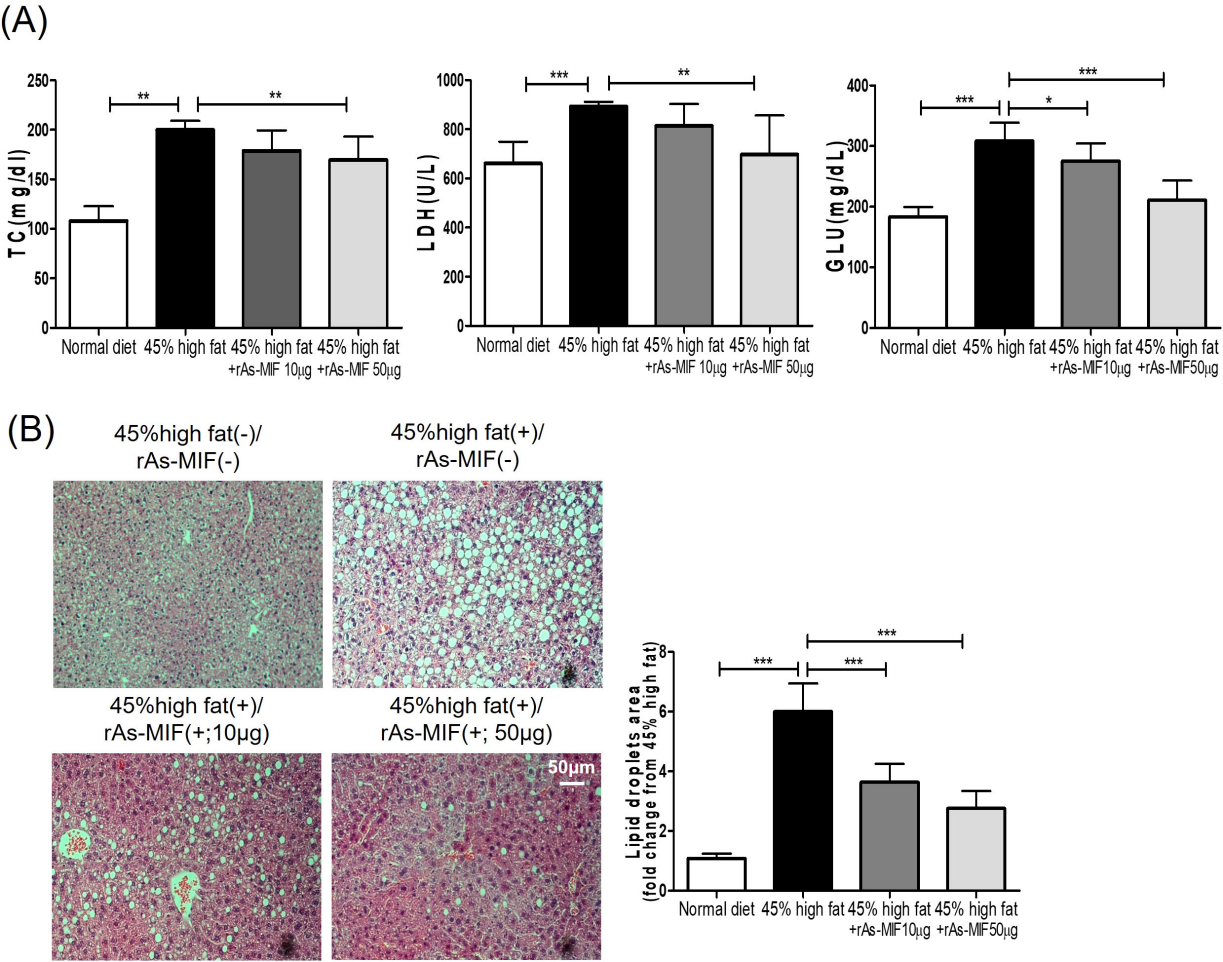
Reduced serum biochemical markers and hepatic adipocytes in 45% HFD-induced obesity by rAs-MIF. **(A)** Serum biochemical indices (TC, LDH, and GLU concentrations). (n = 6/group, 3 independent experiments, *p < 0.05, **p < 0. 01, ***p < 0.001). **(B)** Representative images of liver tissues H&E staining.

Oral administration of rAs-MIF to 45% HFD-fed mice resulted in significant reductions in serum TC and LDH levels compared to only 45%HFD-fed mice (**Figure 4A**). Histological analysis of liver tissues further revealed a marked decrease in both the number and size of lipid droplets in the rAs-MIF-treated group, indicating reduced hepatic lipid accumulation (**Figure 4B**). These findings suggest that rAs-MIF mitigates hyperlipidemia and hepatic steatosis, likely by modulating systemic lipid metabolism and alleviating cellular stress, as reflected by the lower LDH levels.

### 3.5 rAs-MIF reduces obesity-induced inflammatory cytokine production in mice

**Figure 5:**
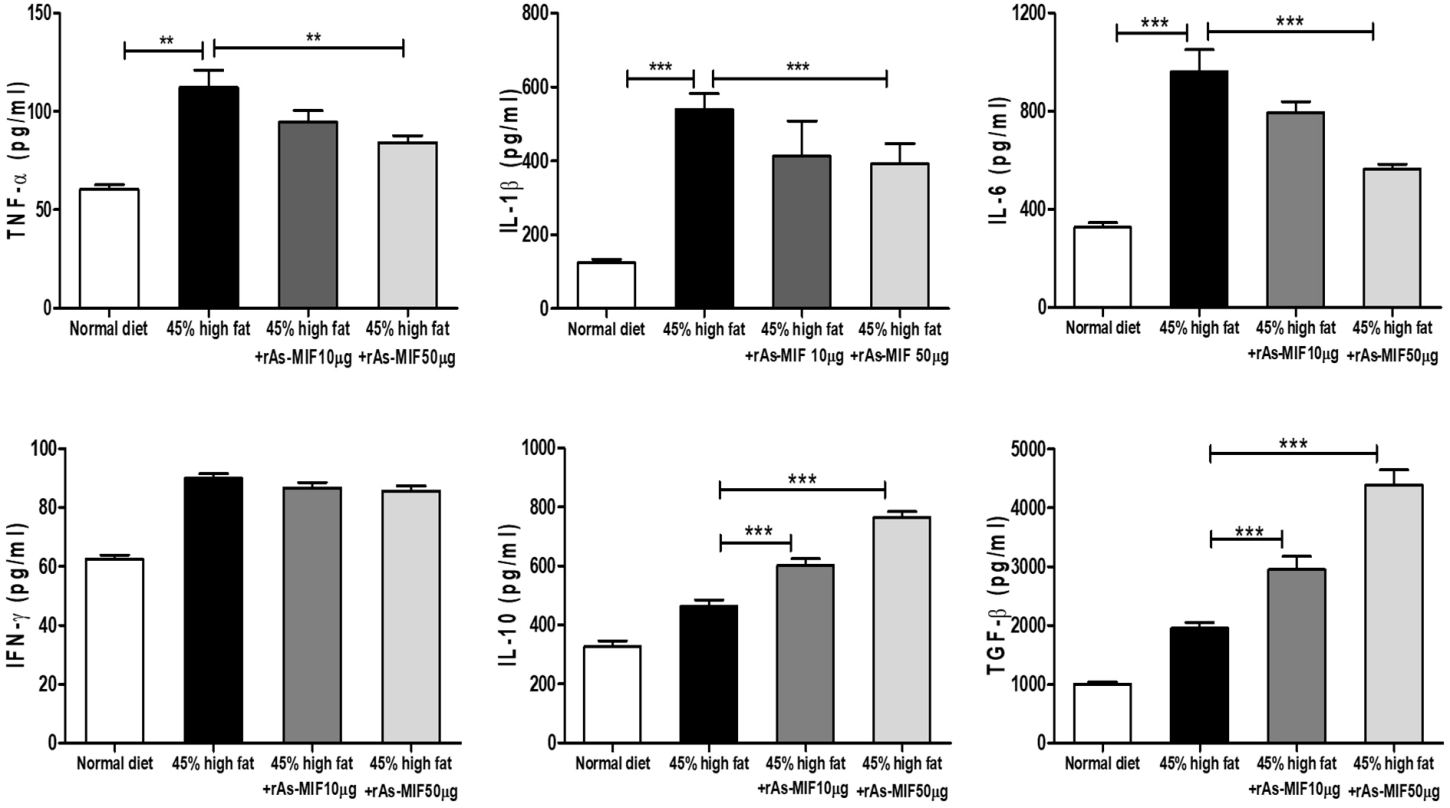
Modulation of serum inflammatory or anti-inflammatory cytokine production levels by rAs-MIF. Serum concentrations of T helper cell-derived cytokines TNF-α, IL-1β, IL-6, IFN-γ, IL-10, and TGF- β were determined using ELISA kits following the manufacturer’s instructions. (n = 6/group, 3 independent experiments, *p < 0.05, **p < 0. 01, ***p < 0.001).

Following the induction of obesity using a 45% HFD for 4 weeks, oral administration of rAs-MIF for an additional 4 weeks significantly reduced the levels of pro-inflammatory cytokines, including TNF-α, IL-1β, and IL-6, in serum compared to the only HFD-fed group (**Figure 5**). Conversely, the expression of anti-inflammatory cytokines IL-10 and TGF-β, associated with regulatory T cell activation, was significantly increased (**Figure 5**). These results suggest that rAs-MIF attenuates obesity-induced inflammation by downregulating the production and circulating levels of inflammatory cytokines.

### 3.6 rAs-MIF restores HFD-triggered immune imbalance by promoting M2 macrophage polarization

**Figure 6:**
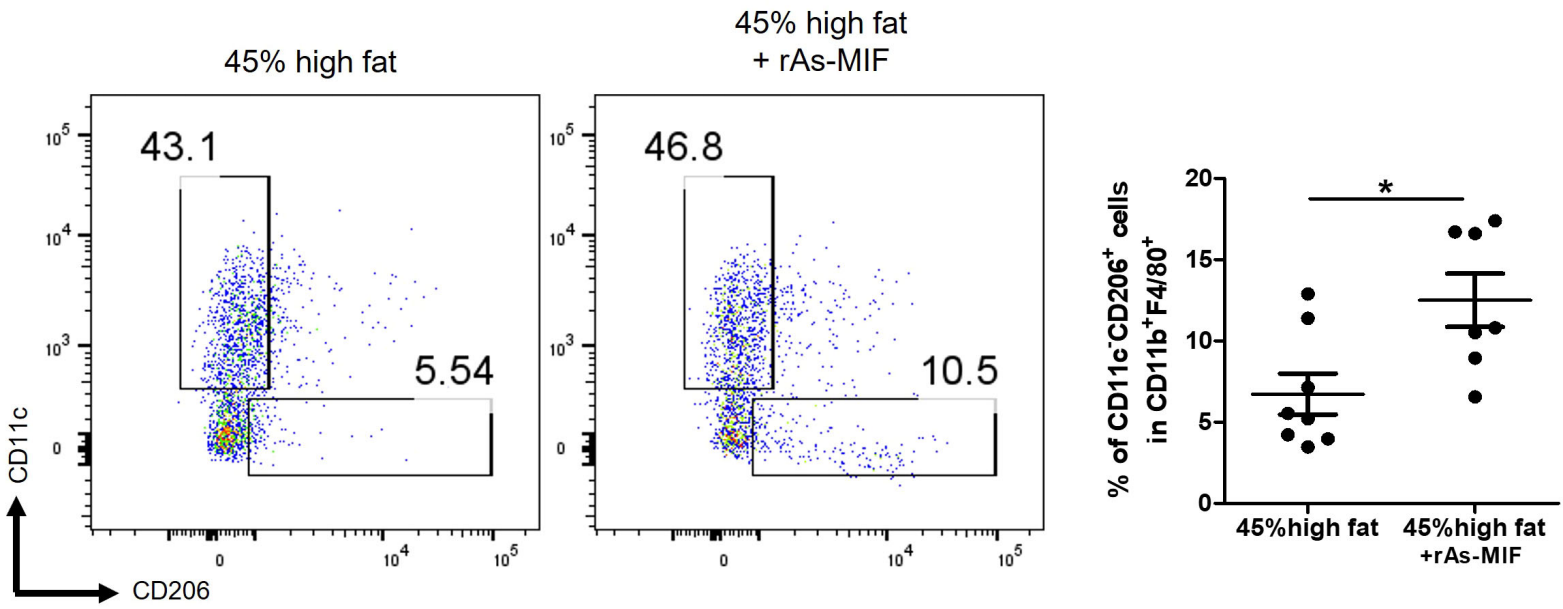
Differences in M1(F4/80^+^CD11c^+^ in F4/80^+^) and M2(CD206^+^ in F4/80^+^) cell populations in adipose tissue induced by rAs-MIF. The isolated cells in eWAT from mice were stained with FITC-anti-mouse F4/80, PE-anti-mouse CD11c, and APC-anti-mouse CD206 antibodies and were analyzed by Flow cytometry. (n = 6/group, 3 independent experiments, *p < 0.05, ***p < 0.001).

To investigate the effects of rAs-MIF on immune modulation within adipose tissue in an obesity-induced model, we analyzed macrophage polarization using flow cytometry in fat tissue. Our results showed that the HFD-only group showed a significant increase in the population of CD11c^+^ in F4/80^+^ M1 macrophages, alongside a decrease in CD206^+^ in F4/80^+^ M2 macrophages, compared to the normal diet control group (**Figure 6**). In contrast, rAs-MIF-treated groups exhibited a reversal of these trends, with a marked decrease in pro-inflammatory M1 macrophages and an increase in anti-inflammatory M2 macrophages (**Figure 6**). Based on the results, the M2/M1 macrophage ratio was calculated and found to have increased by more than twofold following rAs-MIF treatment (**Figure 6**). Furthermore, treatment with rAs-MIF significantly elevated the expression levels of Macrophage Galactose-Type Lectin 2 (MGL2) and CD206 in eWAT, as shown in **Figure 3A**. These results suggest that rAs-MIF can restore immune balance within adipose tissue by promoting M2 macrophage polarization and reducing M1-mediated inflammation. Increased MGL2 and CD206 expression correlated with decreased inflammatory cytokine levels, indicating a shift from M1 to M2 macrophage predominance in eWAT. And this modulation suggests potential beneficial effects, including reducing inflammation, improving metabolic function, and enhancing tissue repair within adipose tissue.

## 4 Discussion

Helminth-derived molecules have emerged as promising therapeutic candidates due to their potent immunomodulatory and metabolic benefits in obesity (32, 33, 61), providing a safer pharmacological alternative to live parasite infection. Specifically, antigens from *Trichinella* sp*iralis* have demonstrated anti-obesity potential by modulating host immune responses and improving metabolic profiles (2, 5, 34). In this study, the present study aimed to elucidate the multifaceted therapeutic efficacy of rAs-MIF, derived from Anisakis simplex. Our results demonstrated that rAs-MIF effectively suppresses obesity by suppressing adipogenesis, promoting M2 macrophage polarization, and restoring systemic metabolic homeostasis.

The differentiation of 3T3-L1 preadipocytes into mature adipocytes serves as a cornerstone model for elucidating the molecular mechanisms governing adipogenesis (3, 35). This complex process is primarily orchestrated by a hierarchical cascade of transcription factors, where peroxisome proliferator-activated receptor gamma (PPARγ) and CCAAT/enhancer-binding protein alpha (C/EBPα) function as the terminal regulators. Previous studies have established that the ectopic expression of PPARγ is sufficient to initiate the adipogenic program in non-adipogenic fibroblasts, whereas PPARγ-deficient embryonic fibroblasts completely lack the capacity for adipocyte differentiation (11, 36). Similarly, C/EBPα plays a critical role in regulating adipogenesis. The ectopic expression of C/EBPα has also been demonstrated to promote adipocyte differentiation, highlighting its importance in the adipogenic transcriptional network (11, 37). In addition to these master regulators, adipokines such as leptin and aP2 (FABP4) play essential roles in adipocyte physiology. Leptin, a hormone primarily secreted by adipocytes, regulates appetite and systemic energy balance via hypothalamic signaling, whereas its chronic overexpression under obese conditions can exacerbate adipose inflammation. Likewise, aP2 facilitates intracellular fatty acid transport and lipid droplet maturation, and elevated expression of aP2 has been associated with insulin resistance and macrophage activation within adipose tissue (38, 39).

In this study, rAs-MIF effectively suppressed the expression of these transcription factors, resulting in decreased lipid droplet accumulation and triglyceride content in 3T3-L1 cells (**Figure 1**). Comparable modulation of adipogenic transcriptional programs has also been reported for other helminth-derived proteins with immunoregulatory properties (40, 41), supporting the concept that parasite molecules can reprogram host metabolic pathways.

The 3T3-L1 cell line, used in the present study, is a well-established murine preadipocyte model that provides high reproducibility and has been widely utilized for mechanistic studies of adipogenesis and lipid metabolism (42–44). However, we acknowledge that this model has limited physiological relevance to human adipose biology. Future investigations will aim to validate the observed effects of rAs-MIF using primary adipocytes or human-derived adipogenic cell lines to confirm the translational potential of these findings.

Beyond its inhibitory role in adipogenesis, rAs-MIF appears to enhance metabolic homeostasis by augmenting the expression of adiponectin, a critical adipokine that modulates glucose and lipid metabolism. The upregulation of adiponectin is known to activate the AMPK (AMP-activated protein kinase) signaling cascade, which catalyzes a metabolic shift toward increased mitochondrial fatty acid oxidation and the induction of uncoupling protein 1 (UCP-1)-mediated thermogenesis (10, 45). Collectively, these data support a model in which rAs-MIF redirects adipose tissue function from energy sequestration to oxidative expenditure via the adiponectin–AMPK–UCP1 axis, highlighting its therapeutic potential as an anti-obesity agent.

*In vivo*, rAs-MIF administration to HFD-induced obese mice significantly reduced body weight gain, fat mass, and serum cholesterol levels while improving glucose tolerance (**Figure 2**). These effects were accompanied by an enhanced FER, reflecting metabolic efficiency. rAs-MIF influenced adipose tissue dynamics by reducing lipid accumulation in eWAT and enhancing thermogenesis in iBAT. Transcriptional analysis revealed upregulation of UCP-1 and PPARα in iBAT and suppression of lipid synthesis genes in eWAT (**Figure 3**), suggesting a shift from energy storage to expenditure. Adipose tissue exhibits depot-specific metabolic and transcriptional characteristics that determine its response to metabolic stimuli. In WAT, transcription factors such as PPARγ and C/EBPα act as master regulators of adipogenesis, promoting triglyceride accumulation and the expression of adipokines such as leptin and aP2, which contribute to lipid storage and inflammatory signaling (46, 47). Accordingly, the downregulation of PPARγ, C/EBPα, and leptin observed in eWAT following rAs-MIF administration likely reflects a suppression of adipogenic and pro-inflammatory transcriptional programs, leading to reduced lipid accumulation and attenuation of adipocyte hypertrophy. In contrast, BAT displays a metabolically active phenotype characterized by high mitochondrial content and thermogenic capacity. In this depot, PPARγ cooperates with coactivators such as peroxisome proliferator-activated receptor gamma coactivator 1-alpha (PGC-1α) to promote lipid oxidation and thermogenic gene expression, including UCP-1 (48, 49). Thus, the upregulation of PPARγ and related metabolic markers in iBAT following rAs-MIF treatment likely represents an adaptive enhancement of energy expenditure rather than lipid storage. This tissue-specific divergence suggests that rAs-MIF mediates a functional reprogramming of adipose depots suppressing adipogenesis and inflammatory signaling in WAT while activating oxidative and thermogenic pathways in BAT to restore metabolic balance under high-fat diet conditions.

While the present study did not incorporate a concurrent pharmacological positive control, the metabolic efficacy of rAs-MIF can be contextualized through established literature benchmarks in diet-induced obesity (DIO) rodent models. Biologically significant weight loss in these models is typically defined by a reduction of ≥10–15% compared to untreated HFD-fed controls (50–52). By aligning with these documented therapeutic thresholds, the observed effects of rAs-MIF provide compelling evidence of its potential as a bioactive agent for managing metabolic dysfunction. Further, rAs-MIF attenuated systemic hyperlipidemia and hepatic steatosis by reducing serum total cholesterol (TC) and lactate dehydrogenase (LDH) levels, along with hepatic lipid droplet accumulation (**Figure 4**). These improvements in lipid metabolism highlight the potential of rAs-MIF to alleviate metabolic complications associated with obesity.

M2 macrophages are essential for maintaining adipose tissue homeostasis by secreting anti-inflammatory cytokines, such as IL-10 and TGF-β, which improve insulin sensitivity and mitigate fibrosis. Our findings demonstrate that rAs-MIF restored immune balance by enhancing M2 macrophage polarization and suppressing M1 macrophage activity in eWAT, as evidenced by flow cytometry and increased expression of M2 markers such as CD206 and MGL2 (**Figure 6**). The anti-inflammatory activity of rAs-MIF may be mediated through the IL-4/IL-13–STAT6–PPARγ signaling cascade, which promotes M2 polarization and the production of regulatory cytokines such as IL-10 and TGF-β (53–55). This contrasts with mammalian MIF, which supports M1 activation through NF-κB and MAPK signaling (56, 57). The divergent roles of rAs-MIF underscore the adaptive immunomodulatory strategies employed by helminths to promote survival while mitigating host inflammation (1, 24, 58).

Chronic inflammation is a hallmark of metabolic dysfunction associated with obesity. In the present study, rAs-MIF significantly reduced serum levels of pro-inflammatory cytokines, including TNF-α, IL-1β, and IL-6, while concomitantly promoting anti-inflammatory cytokines IL-10 and TGF-β (**Figure 5**). Although several studies have reported reduced circulating IL-10 levels in obesity, accumulating evidence indicates that the expression of anti-inflammatory cytokines such as IL-10 and TGF-β can be elevated in a tissue-specific manner following chronic high-fat feeding. This phenomenon likely represents a compensatory immune regulatory response within adipose tissue, aimed at counterbalancing sustained pro-inflammatory signaling and facilitating tissue remodeling. Indeed, previous studies have demonstrated increased IL-10 and TGF-β expression in adipose tissues of HFD-fed or genetically obese mice, underscoring that local cytokine profiles do not necessarily parallel systemic inflammatory patterns (59, 60). In this context, rAs-MIF restores immunometabolic balance within adipose tissue by coordinating these immune and metabolic processes, highlighting its therapeutic potential for obesity and related metabolic disorders.

Collectively, these findings demonstrate that rAs-MIF and other parasite-derived molecules are promising candidates for obesity management by targeting adipogenesis, energy metabolism, and immune regulation. Furthermore, future research should focus on isolating the bioactive compound, optimizing formulation and delivery strategies, and validating the long-term safety and efficacy of rAs-MIF in clinical applications.

## Data Availability

The data that support the findings of this study are available from the corresponding author upon reasonable request.
